# Polyphenols: Immunomodulatory and Therapeutic Implication in Colorectal Cancer

**DOI:** 10.3389/fimmu.2019.00729

**Published:** 2019-04-11

**Authors:** Anna Maria Mileo, Paola Nisticò, Stefania Miccadei

**Affiliations:** Tumor Immunology and Immunotherapy Unit, Department of Research, Advanced Diagnostic and Technological Innovation, IRCCS Regina Elena National Cancer Institute, Rome, Italy

**Keywords:** polyphenols, immunomodulation, inflammation, gut microbiota, colorectal cancer, colorectal cancer therapy

## Abstract

Polyphenolic compounds, widely present in fruits, vegetables, and cereals, have potential benefits for human health and are protective agents against the development of chronic/degenerative diseases including cancer. More recently these bioactive molecules have been gaining great interest as anti-inflammatory and immunomodulatory agents, mainly in neoplasia where the pro-inflammatory context might promote carcinogenesis. Colorectal cancer (CRC) is considered a major public healthy issue, a leading cause of cancer mortality and morbidity worldwide. Epidemiological, pre-clinical and clinical investigations have consistently highlighted important relationships between large bowel inflammation, gut microbiota (GM), and colon carcinogenesis. Many experimental studies and clinical evidence suggest that polyphenols have a relevant role in CRC chemoprevention, exhibit cytotoxic capability vs. CRC cells and induce increased sensitization to chemo/radiotherapies. These effects are most likely related to the immunomodulatory properties of polyphenols able to modulate cytokine and chemokine production and activation of immune cells. In this review we summarize recent advancements on immunomodulatory activities of polyphenols and their ability to counteract the inflammatory tumor microenvironment. We focus on potential role of natural polyphenols in increasing the cell sensitivity to colon cancer therapies, highlighting the polyphenol-based combined treatments as innovative immunomodulatory strategies to inhibit the growth of CRC.

## Introduction

Colorectal cancer is the world's second deadliest cancer after lung (1), more than half (55%) of the cases of CRC occur in developed regions, but developing countries are catching up. As their economies grow, so does the incidence of colorectal cancer. The scientific data show that no correct dietary habits and life style may affect the risk/progression of inflammation-related disease such as CRC (2–8). In inflammatory bowel disease (IBD) chronic inflammation leads to mucosal disruption accompanied by an excessive production of reactive oxygen species (ROS) (9) and may promote cancer onset, progression and metastatic diffusion (10). Apart from IBD, inflammation-related cancer risk factors are high fat/low fiber diet, obesity, and family history of CRC, all linked to an abnormal gut microbial composition (11–14). In sporadic (no-colitis-associated) CRC genetic alterations, such as adenomatous polyposis coli (Apc) mutations, DNA mismatch repair (MMR) deficiency, and microsatellite instability (MSI) are reported (15). Immune microenvironment of MSI primary colon cancer revealed a high infiltration of activated CD8+ CTL (16) and high expression of immune checkpoints, indicating that this subgroup of tumors may represent a target of immune checkpoint blockade (ICB). These results stem from seminal data by Galon et al. demonstrating that the lymphocyte infiltration in the primary CRC is a good prognostic factor (17). Clinical and experimental studies have focused on the complex and essential role of immune responses in CRC. In this scenario the crucial role of gut microbiota in triggering chemokine production leading to T cell recruitment in tumor tissues has been demonstrated (18). Notably dietary bioactive compounds act as anti-tumor agents (19, 20) modulating several molecular targets involved in survival, proliferation, angiogenesis, invasion and metastasis in cancer (21–23). As such several nutrients (24) may affect crucial inflammatory chemokines and cytokines which strongly contribute to the epithelial-mesenchymal transition program involved in cancer invasion, metastasis, and immune escape process (25) also in CRC (26).

**Figure 1:**
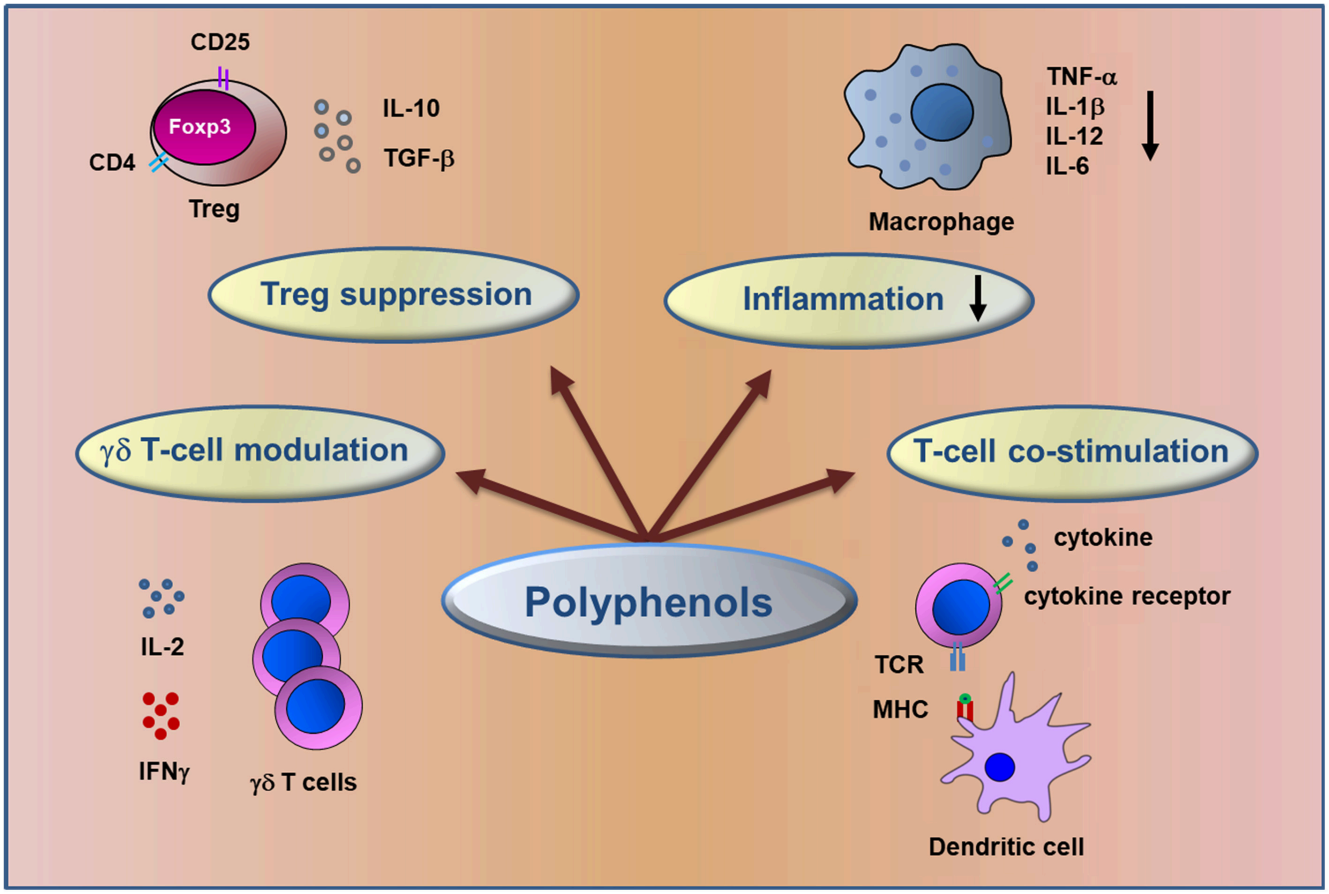
Schematic representation of immunomodulatory action of dietary polyphenols. The figure summarizes the major mechanisms involved in the control of inflammation and in anti-tumor immune response elicited by polyphenols in CRC.

Among bioactive compounds, polyphenols are known as potential anti-inflammatory and anti-tumor agents (Figure 1) and could be good candidates for cancer prevention and treatment (27–29) targeting key molecular pathways involved in colorectal cancer (30–32).

Epidemiological studies suggest that the Mediterranean Diet *(*MD) rich in polyphenols, could reduce level of inflammation and oxidative damage and has linked with a relevant reduction in cancer risk mainly in CRC (33, 34).

Considering the role of inflammation in CRC, anti-inflammatory agents such as polyphenols are promising drugs for less toxic clinical intervention. In this review we detail the polyphenol properties, most likely related to their immunomodulatory activities, able to counteract several aspects of the inflammatory process in tumor microenvironment through the modulation of cytokine and chemokine production and in turn immune cell activation (35).

## Dietary Polyphenols

Polyphenols are a large group of compounds synthesized by plants for a variety of functions, such as protection against UV radiation, mechanical damage, and microbial infection (36). Different classes and subclasses of polyphenols generate a large structural variability as a function of their number of phenol rings in combination with one or more hydroxyl compounds (37). Polyphenols present in fruits, vegetables, and cereals have emerged as one of the main families of natural compounds, most of them have been considered functional foods with potential biological activities in many pathologies, such as cancer (38–40), diabetes (41), obesity and inflammation-related diseases (42–44), neurodegenerative disorders (45, 46), and cardiovascular diseases (47). The main groups of dietary polyphenols promoting beneficial effects on human health are: phenolic acids, flavonoids, stilbenes, and lignans (see http://phenol-explorer.eu/compounds/classification for an update classification).

They are potential anti-inflammatory and anti-cancer agents, especially in anatomical districts such as colon, where the activity of dietary bioactive compounds is relevant.

### Cross Talk Between Polyphenols and Gut Microbiota

After oral assumption, dietary polyphenols are recognized by the human body as xenobiotics and only a small amount is hydrolyzed in active compounds and absorbed in the small intestine (48). The remaining polyphenols accumulate in the large intestinal lumen where they might be hydrolyzed by the enzymatic activities of the gut microbial community into various metabolites before absorption. It is becoming clear that the GM, after degradation of food macromolecules (49), plays a crucial role in the maturation, development, and function of the host immune system (18, 50–52). Nevertheless, a continuous dialogue between microbiota and innate and adaptive immunity (53) regulates intestinal tissue homeostasis. When this functional control is lost, dysbiosis occurs leading to inflammatory disorders that might promote tumorigenesis (54–58).

Recently, advances in defining the effects of the microbiota on polyphenol bioavailability and function have been made. Moreover, it has been recognized that polyphenol-rich foods can affect the composition and activity of the microbiota (59, 60). Dietary polyphenols exert a prebiotic-like effect, contribute to maintain the gut health and to reduce levels of inflammation through the stimulation of beneficial bacteria growth and inhibition of pathogenic microbe development (61, 62). The large inter-individual variation of metabolites (30) can be related, at least partly, to differences in the GM composition and therefore in the way the polyphenols are catabolized.

Epidemiological data (33, 63) are concordant in suggesting that the adherence to MD decreases the risk of a variety of cancers. The polyphenol-rich MD modulates multiple processes involved in inflammatory response and carcinogenesis, such as free radical production, nuclear factor kappa-light-chain-enhancer of activated B cells (NF-κB) activation, and the eicosanoid-derived pathways. Thus, an appropriate diet can maintain the equilibrium between the inflammatory pathway and the anti-inflammatory process induced by many dietary compounds including polyphenols (64). Furthermore, great attention has been addressed to the effect that MD has on maintenance of “good” GM (65) and on control of carcinogenesis through specific epigenetic alterations (66).

Recently, the association between microbiota and different stages of CRC and the role of microbiota in the therapeutic response including that of the immune checkpoint-blocking therapy has shown to be highly relevant (67).

### Polyphenols as Immunomodulatory Agents

Growing evidence clearly indicates that diet influences the innate and adaptive arms of the immune system (Figure 1) (68). Innate immune cells, such as macrophages, myeloid-derived suppressor, dendritic and natural killer cells and adaptive immune cells (T and B lymphocytes) may infiltrate the tumor tissues impacting the immune microenvironment and the clinical outcome (49). Several investigations have reported the antitumor effect of polyphenols through the modulation of T lymphocyte functionality to recognize and lyse tumor cells enhancing the immune response and counteracting the immune escape, a cancer progression hallmark (69, 70).

Among polyphenol studies, the dose dependent therapeutic efficacy of resveratrol has been demonstrated in an animal model of ulcerative colitis. Resveratrol, a flavonoid stilbene compound, treatment is associated with the regulation of T regulatory (Treg)/T helper 17 (Th17) balance and the level of plasma and intestinal mucosal cytokines including interleukin 10 (IL-10), transforming growth factor-beta1 (TGF-β1), interleukin 6 (IL-6), and interleukin 17 (IL-17) (71).

Curcumin, a no-flavonoid compound, suppressed the development of Dextran Sulfate Sodium (DSS)-induced colitis in a mouse model through the inhibition of NF-κB activation and the induction of mucosal Treg cells. Treatment with nanoparticles of curcumin induced an alteration of the gut microbial structure and a change in the level of fecal short chain fatty acids, indicating nanoparticles curcumin as a therapeutic option for the treatment of IBD diseases (72).

*Trans*-Scirpusin A (TSA), a resveratrol oligomer, has been reported to inhibit the growth of colorectal cancer in *in vivo* model. Among different mechanisms involved in its antitumor efficacy, TSA overcomes the tumor-associated immunosuppressive microenvironment by reducing the number of CD25-FoxP3 regulatory T cells and myeloid-derived suppressor cells (73).

Dietary anti-inflammatory polyphenols, such as chlorogenic acid and resveratrol, significantly suppressed the secretion of several cytokines (interferon gamma IFN-γ, tumor necrosis factor alfa TNF-α), reduced the colonic infiltration of CD3^+^T cells producing these cytokines and neutrophils in colitis and in colitis-associated to CRC experimental model (74, 75). In mouse C57BL/6J-Min/^+^, bearing a germline Apc mutation, a classical model of familial adenomatous polyposis (FAP) and sporadic colorectal cancer, administration of curcumin increases mucosal CD4^+^ T and B cells and contributes to prevent adenoma formation (76). Apple polyphenolic extracts (77), soy and sulforaphane flavonoid compounds (78, 79) reduce pro-inflammatory cytokine expression (IL-1β, TNF-α, IL-6, IL-17, and IFN-γ) in the context of a chemically induced colitis. The immunomodulatory activity of cocoa, a flavonoid compound, has been demonstrated to affect the gut immune responses in young rats by increasing the percentage of γδ T cells and lowering the effect of IgA (80).

It has been demonstrated an important role of chemokine receptor 3 (CXCR3) and its ligands in both inflammation and colorectal cancer (81). Recently it has been shown that resveratrol trihydroxy trans stilbene (TS) is able to hamper the suppressive ability of Treg by increasing CXCR3 in CD8+ Tregs facilitating Teff recruitment with a reduction of number and size of tumors (82). Dendritic cells (DCs) play a relevant role in cancer by exerting both pro-tumorigenic and anti-tumorigenic functions depending on the local environment (83) having an impact upon clinical outcome in CRC patients (84). Furthermore, Kajihara et al. analyzed the potential role of a dendritic-based cancer immunotherapy treatment in patients with recurrent or metastatic CRC (85). The potential effects of polyphenols on the function of DCs, the most effective antigen-presenting cells, with the capacity to shift immune response toward tolerance or immune activation (86, 87) has been highlighted in different studies. In particular protocatechuic acid, an anthocyanin flavonoid metabolite, has been demonstrated to have a main regulatory effect on the functional activation of DCs, suggesting the potential therapeutic application of this dietary compound against inflammatory conditions (88).

Quercetin and piperine, a flavonoid and no-flavonoid compound, when combined represent an effective and potent anti-inflammatory strategy to treat acute colitis in mice. This anti-inflammatory effect was mediated by impaired DC immune responses (89). Moreover, quercetin has been shown to suppress the secretion of TNF-α and interfere with the onset of IBD (90).

### Polyphenols and Inflammation

Chronic inflammation characterized by the continuous presence of inflammatory cells and inflammatory mediators is known to play a key role in the development of various cancers. A long-standing chronic inflammation is a key predisposing factor of CRC in IBD since an excessive ROS production present in the inflamed mucosa alters important cellular functions and damages intestinal mucosa (91–93).

For several years, polyphenols have been extensively studied for their ability to scavenge free-radicals endogenously generated (94) or formed by radiation and xenobiotics (95).

Polyphenols have anti-inflammatory effect and their antioxidant properties are mainly mediated by ability to down-regulate the nuclear factor NF-*k*B, modulating crucial cell signaling pathways involved in inflammation and cancer (96–100).

Evaluating the polyphenolic fraction of olive oil, a principal component of MD, Serra et al. reported the inhibition of some crucial colonic inflammatory processes mediated by NF-*k*B, inducible nitric oxide synthases (iNOS), IL-8 and IL-6, thus proposing olive oil as a major dietary compound able to prevent and counteract the progression of IBD (101).

Green tea polyphenols ameliorate antioxidant response, decrease inflammatory markers (TNF-α, IL-6, and serum amyloid A), attenuate the pathological lesions and preserve colonic microstructure in a similar manner of sulfasalazine, the standard-of-care agent in IBD as shown in DSS mice (102). Several studies have demonstrated that cocoa exhibits a potential anti-inflammatory effects in *in vitro* and *in vivo* CRC models and might be a promising dietary preventive compound. Experiments performed in TNF-α stimulated colon cancer Caco-2 cells demonstrated that cocoa polyphenols down-regulate inflammatory marker expression by suppressing NF-*k*B nuclear translocation and c-Jun N-terminal kinase (JNK) phosphorylation. A cocoa-rich diet decreased the nuclear level of NF-*k*B and the expression of cyclooxigenase-2 (COX-2) and nitric oxide (NO) synthase pro-inflammatory enzymes, in a colon carcinogenic rat model (98, 99). Moreover, recent findings demonstrated that cocoa suppresses the development of colitis-associated tumorigenesis by modulating NF-*k*B/IL-6/Signal transducer and activator of transcription 3 (STAT3) signaling pathways and also induces apoptosis by activating caspase-3 in a mouse model (103).

Evidence has been provided that pomegranate phenolic extract (PE) and/or its microbiota derived metabolite-urolithin-A (UROA), have antioxidant, anti-inflammatory, and anticancer properties as showed by Larrosa et al. (104). They demonstrated that pomegranate could prevent colon inflammation before and during colitis disease in rats. PE and UROA decreased inflammation markers (*i*NOS, COX-2, prostaglandin E synthase, and prostaglandin E2) in colonic mucosa and could have a beneficial effect on the gut microbiota.

Flavanol extract from *Chaenomeles japonica*, a Japanese fruit, inhibited COX-2, matrix metalloproteinase-9 (MMP-9), and NF-*k*B expression indicating a cytotoxic anti-inflammatory and anti-metastatic activities toward the colon cancer SW-480 cells (105).

A recent study has shown that sugarcane extracts, containing a potent reservoir of polyphenols, provide anti-inflammatory activity by decreasing NF-*k*B phosphorylation and subsequently reduced expression of IL-8 signaling in lipopolysaccharide-stimulated colon cancer cells (106).

Edible mushroom (*Pleurotus eryngii*), rich in polyphenols, has been shown to inhibit the production of pro-inflammatory molecules, such as ROS and NO in RAW264.7 macrophage cell line and to induce an anti-proliferative effect on HCT116 colon cancer cells (107). All these results indicate the relevance of dietary polyphenol intake derived from food throughout the world.

The consumption of many varieties of plant derivatives is very ancient remedy against all kinds of ailments. Since ancient times folk medicine has made use of plants like *Opuntia ficus indica* and *Ceratonia Siliqua* and recently their polyphenolic extracts have received much attention for their antioxidant, anti-inflammatory, immunomodulatory and anticancer properties. Aboura et al. demonstrated the protective effects of polyphenol-rich infusions from *C. Siliqua* leaves on inflammation either in *in vitro* and *in vivo* experimental models, reducing pro-inflammatory cytokine expression (such as TNF-α, IL-1β, and IL-6), and NO production (108). The anti-inflammatory activity of *Garcinia Kola* (109) and *Ocimum Gratissimum* (110) polyphenolic extracts is exerted by reducing TNF-α and pro-inflammatory cytokine production in a rat colitis experimental model.

### Potential Role of Polyphenols in CRC Prevention and Therapy

Decades of research on bioactive compounds have highlighted the beneficial effects of anti-inflammatory polyphenols not only in preventing cancer, but also in potentiating the efficacy of chemo/radiotherapy and reducing the risk of tumor recurrence (111).

Primary prevention of colon cancer, using dietary agents, boasts a widespread use in healthy populations (112) and in IBD patients at high risk of developing CRC (113).

Protective effects of apple and berry fruits against colon cancer have been demonstrated in *in vivo* studies. The Apc ^Min+^ mouse model has been used to show the chemopreventive effects of Annurca apple polyphenol extract, which decreased polyp numbers and size, in animals fed a balanced-diet and also counteracted the tumorigenic effect of a diet rich in fats (114).

The chemopreventive effect of black raspberries (BRB) in DSS-mice was mediated by the downregulation of the pro-inflammatory cytokine expression including TNF-α and IL-1β (115) and the decrease of COX-2 and plasma prostaglandin E2 levels. Furthermore, Pan et al. performed a comprehensive analysis of BRB metabolic profiles correlated with a decrease of polyp number and size in Apc ^Min+^ mice (116).

Consistent with the well-known antioxidant and anti-inflammatory properties of resveratrol (117) Cui et al. reported a decrease of colon inflammation score through the down regulation of mucosal and/or systemic TNF-α and IFN-γ expression in a mouse model of colitis (75). IL-10-deficient mouse, a well-documented model for colitis-associated cancer, was used to investigate the chemopreventive properties of curcumin which has been shown to have anti-inflammatory, anti-oxidative and anti-proliferative properties. The chemopreventive effect of curcumin reduced colonic tumor burden and was associated with the maintenance of a high microbial diversity (118).

Polyphenols may also behave as pro-oxidants (119, 120), triggering ROS-mediated cancer cell death (121, 122), affecting tumor cell behavior based on differential redox status of cancer cells comparing with normal ones (123). Cocoplum (*Chrysobalanus icaco* L.) anthocyanin-rich tropical fruit exerts anti-inflammatory activity through the reduction of TNF-α, IL-1β, IL-6, and NF-κB expressions and pro-oxidant effect on HT-29 human colorectal adenocarcinoma cells (124).

Conventional radio/chemotherapeutic treatment may induce immunogenic cell death (ICD), essential for activation of a T cell dependent immune response specific for dead cell-derived antigens. The improved efficacy of cancer therapy when tumor cells undergo an ICD (125–127) leads to the stimulation of T cells by DC through capture, processing, and presentation of antigens to naïve CD4^+^ and CD8^+^ T cells, which in turn elicit an antitumor response (128). In CRC patients clinical applications of therapeutic drugs have been greatly restricted due to the acquired chemoresistance as well as side-effect toxicity (129). These limitations highlight the need for development of novel anti-tumor strategies that may enhance chemosensitivity of tumor cells and reduce toxicity (130).

The concept of combined therapy of anti-cancer drugs with natural compounds has become a very promising approach in designing effective clinical trials. Evidence is emerging that conventional chemotherapy in CRC significantly benefits through combined treatment with some natural dietary polyphenols (131–135). Indeed several studies have demonstrated the effective and enhanced anticancer activity of 5-Fluorouracil (5-FU) in combination with natural compounds and suggested the possibility of reducing dose-related toxicity and resistance to this drug (136, 137). This effect has been demonstrated by Hakim et al. who combined treatment of Gelam honey and ginger (*Zingiber officinale*) phenolic acid rich-extracts to enhance the chemotherapeutic effect of 5-FU against a colon adenocarcinoma cell line (138).

In recent years several studies have demonstrated the presence and the relevance of cancer stem cells (CSC) in different tumors including CRC (139–141). These cells are hypothesized to be responsible for tumor relapse and resistance to conventional therapies. Novel anticancer strategies have been designed to selectively target CSC and in this scenario natural polyphenols might have a relevant role.

Shakibaei et al. illustrated interesting effects of curcumin in enhancing chemosensitization to 5-FU-based chemotherapy by targeting the CSC subpopulation (142). Dietary bioactive polyphenolic metabolites derived from pomegranate and berries reduced the number and size of CSC colonspheres and modulated chemotherapy resistance (143).

Given that gastrointestinal cancer cells are poorly responsive to ionizing radiation treatment, novel radiosensitizer agents are required to improve the efficacy of CRC radiotherapy. Fisetin, a dietary flavonoid, has been demonstrated to enhance the radiosensitivity of p53-mutant HT-29 human colorectal cancer cells by increasing radiation induced DNA double strand breaks (144).

## Conclusions

The current review reports recent epidemiological and experimental data supporting the bright future of dietary polyphenols as chemopreventive, anti-inflammatory, immunomodulatory, and anticancer agents in CRC (Figure 1). The polyphenol-rich diet not only may represent a chemopreventive treatment but also has important function on immune system by promoting symbiont and commensal bacterial populations, increases reciprocal interaction between host and microbiota which in turn have important effects on immune function Evidence underlines the use of polyphenols as sensitizers of chemo/radiotherapies paving the way for new combined strategies able to minimize toxicity and side effects of conventional treatment in oncological patients. In the new era of precision medicine it would be important to consider the oxidative status prior to polyphenol supplementation or dietary advice. Recent advance in metabolomics might reveal individual oxidative bio-signatures of cancer patients leading to a personalized treatment approach.

Further studies are needed to deeply evaluate the immunomodulatory role of polyphenols to improve the efficacy of novel treatment such as immune checkpoint blockade, a new reality in oncology.

## Author Contributions

SM and AM have made a substantial, direct and intellectual contribution to the article designing. PN contributed to the writing and revised the article. All authors have reviewed and approved the final version of the manuscript for publication.

### Conflict of Interest Statement

The authors declare that the research was conducted in the absence of any commercial or financial relationships that could be construed as a potential conflict of interest.
